# Establishment and validation of an endoplasmic reticulum stress reporter to monitor zebrafish ATF6 activity in development and disease

**DOI:** 10.1242/dmm.041426

**Published:** 2020-01-28

**Authors:** Eric M. Clark, Hannah J. T. Nonarath, Jonathan R. Bostrom, Brian A. Link

**Affiliations:** Department of Cell Biology, Neurobiology and Anatomy, Medical College of Wisconsin, Milwaukee, WI 53214, USA

**Keywords:** ATF6, Endoplasmic reticulum stress, Unfolded protein response, Zebrafish, Neurodegeneration

## Abstract

Induction of endoplasmic reticulum (ER) stress is associated with diverse developmental and degenerative diseases. Modified ER homeostasis causes activation of conserved stress pathways at the ER called the unfolded protein response (UPR). ATF6 is a transcription factor activated during ER stress as part of a coordinated UPR. ATF6 resides at the ER and, upon activation, is transported to the Golgi apparatus, where it is cleaved by proteases to create an amino-terminal cytoplasmic fragment (ATF6f). ATF6f translocates to the nucleus to activate transcriptional targets. Here, we describe the establishment and validation of zebrafish reporter lines for *ATF6* activity. These transgenic lines are based on a defined and multimerized *ATF6* consensus site, which drives either eGFP or destabilized eGFP, enabling dynamic study of *ATF6* activity during development and disease. The results show that the reporter is specific for the ATF6 pathway, active during development and induced in disease models known to engage UPR. Specifically, during development, *ATF6* activity is highest in the lens, skeletal muscle, fins and gills. The reporter is also activated by common chemical inducers of ER stress, including tunicamycin, thapsigargin and brefeldin A, as well as by heat shock. In models for amyotrophic lateral sclerosis and cone dystrophy, *ATF6* reporter expression is induced in spinal cord interneurons or photoreceptors, respectively, suggesting a role for ATF6 response in multiple neurodegenerative diseases. Collectively our results show that these *ATF6* reporters can be used to monitor *ATF6* activity changes throughout development and in zebrafish models of disease.

This article has an associated First Person interview with the first author of the paper.

## Abstract (precis)

**Summary:** In this study, we validate transgenic zebrafish generated to specifically report the activity of ATF6, representing a major branch of the endoplasmic reticulum stress pathway with functions in development and disease.

## INTRODUCTION

The endoplasmic reticulum (ER) is an important organelle in the cell for biosynthesis, folding and maturation of proteins destined for the cell membrane or the extracellular space (Görlach et al., 2006). The ER also plays other important roles, including lipid biosynthesis (Sriburi et al., 2004), calcium homeostasis (Görlach et al., 2006), and genesis of autophagosomes (Hayashi-Nishino et al., 2009) and peroxisomes (Geuze et al., 2003). Resident to the ER are multiple chaperones, foldases and co-factors to ensure rapid and functional protein production and to protect the ER when translational demand is elevated (Görlach et al., 2006). If the protein load becomes too high, the ER can expand to increase surface area (Schuck et al., 2009), or target misfolded proteins for degradation by the proteasome (Meusser et al., 2005; Stolz and Wolf, 2010) or the lysosome (Ishida and Nagata, 2009).

Another strategy to maintain ER homeostasis in the presence of misfolded proteins or other stress at the ER is transcriptional regulation. This regulation can affect the described mechanisms as well as downregulate overall translation to decrease the protein quantity, while increasing the functional capacity of the ER to promote cell survival. If pro-survival modifications are insufficient, chronic ER stress can upregulate transcription of pro-apoptosis machinery (Walter and Ron, 2011). These pro-survival and pro-apoptosis transcriptional modifications are downstream of three main unfolded protein response (UPR) pathways: ATF6, IRE1 (also known as ERN1) and PERK (also known as EIF2AK3) (Walter and Ron, 2011). In the normal state, ATF6, IRE1 and PERK proteins are tethered at the ER through interaction with BIP/GRP78 (also known as HSPA5) in the lumen. After ER stress, the tethered proteins are released and able to activate the UPR. The mechanisms for activation for each branch of the ER stress response network are conserved among vertebrates, including mammals and fish (Ishikawa et al., 2011).

ATF6 is synthesized as a 90-kDa protein with 670 amino acids. It is a member of the ATF/CREB basic-leucine zipper (bzip) DNA-binding protein family. After ER-stress-induced release of ATF6 from the ER, its Golgi localization signal is exposed. At the Golgi, ATF6 is cleaved by site 1 protease (S1P; also known as MBTPS1) and site 2 protease (S2P; also known as MBTPS2), leaving a 50-kDa cleavage product, ATF6f (Hillary and FitzGerald, 2018). ATF6f translocates to the nucleus and can bind to ER-stress-response elements (Roy and Lee, 1999; Yoshida et al., 1998). Later, Wang et al. (2000) identified the specific consensus binding sequence for ATF6, TGACGTGGCGATTCC. In the current study, this consensus sequence was used to build a reporter for examining *ATF6* activation *in vivo*, which has not been previously monitored.

Recently, ATF6 has been implicated in the development of multiple tissue types, and dysregulation of *ATF6* expression is related to disease (Hillary and FitzGerald, 2018). Developmental roles of ATF6 include mesoderm differentiation (Kroeger et al., 2018), osteogenesis and chondrogenesis (Guo et al., 2016; Jang et al., 2012; Kim et al., 2014; Maeda et al., 2015; Xiong et al., 2015), neurodevelopment (Cho et al., 2009; Naughton et al., 2015; Saito et al., 2012; Zhang et al., 2007), adipogenesis and lipogenesis (Lowe et al., 2012), and formation of female reproductive structures (Lin et al., 2012; Park et al., 2013; Xiong et al., 2016; Yang et al., 2015). Most relevant to our work is the role of ATF6 in ocular and muscular embryology, because these tissues had the highest *ATF6* reporter expression during zebrafish development. *ATF6* is also dynamically expressed throughout lens formation (Firtina and Duncan, 2011). In muscle development, ATF6 functions in apoptosis (Nakanishi et al., 2005) and differentiation (Wang et al., 2015) processes. Dysregulation or loss of ATF6 during development and in adulthood is thought to contribute to neurological disorders. Recently, mutations in multiple domains of *ATF6* were identified in patients with autosomal recessive achromatopsia (Chiang et al., 2017; Kohl et al., 2015). The IRE1/XBP1 pathway has been implicated in retinitis pigmentosa (Chiang et al., 2015), suggesting that investigation of ATF6 and other UPR pathways is important for understanding photoreceptor homeostasis and disease. Multiple neurodegenerative diseases including amyotrophic lateral sclerosis (ALS) have misfolded proteins and signatures of ER stress. A *Sod1 G93A* mouse model of ALS had elevated levels of Atf6f compared to wild-type mice at early symptomatic and end stages of disease, suggesting that Atf6 could play an important role in progression of ALS and other neurodegenerative conditions (Kikuchi et al., 2006).

In zebrafish, Atf6 has been investigated in fatty liver disease and steatosis (Cinaroglu et al., 2011; Howarth et al., 2014), but how Atf6 is involved in other tissue types or disease processes has not been investigated. Although cell stress reporters for monitoring *xbp1* splicing and Atf4 translational induction have been described in several species (Iwawaki et al., 2017; Kang et al., 2015; Li et al., 2015; Ryoo et al., 2013), there are currently no reporters for investigating *ATF6* activity *in vivo* for any animal model. To address this unmet need, we created a zebrafish reporter based on a previously identified *ATF6* consensus site (Wang et al., 2000) to drive either enhanced GFP (eGFP) or destabilized eGFP (d2GFP), enabling dynamic study of *ATF6* activity during development and disease *in vivo*. *ATF6* was activated in multiple tissues throughout development, can be induced by ER stress, and is upregulated in zebrafish disease models of cone dystrophy and ALS. Together, our data show that the *ATF6* reporter is a useful tool for dynamically monitoring *ATF6* activity and could be used in combination with other reporters to investigate the complicated nature of UPR signaling in development and disease progression.

## RESULTS

### Establishment of an ATF6 response element reporter for *in vivo* monitoring of ER stress

**Fig. 1.**
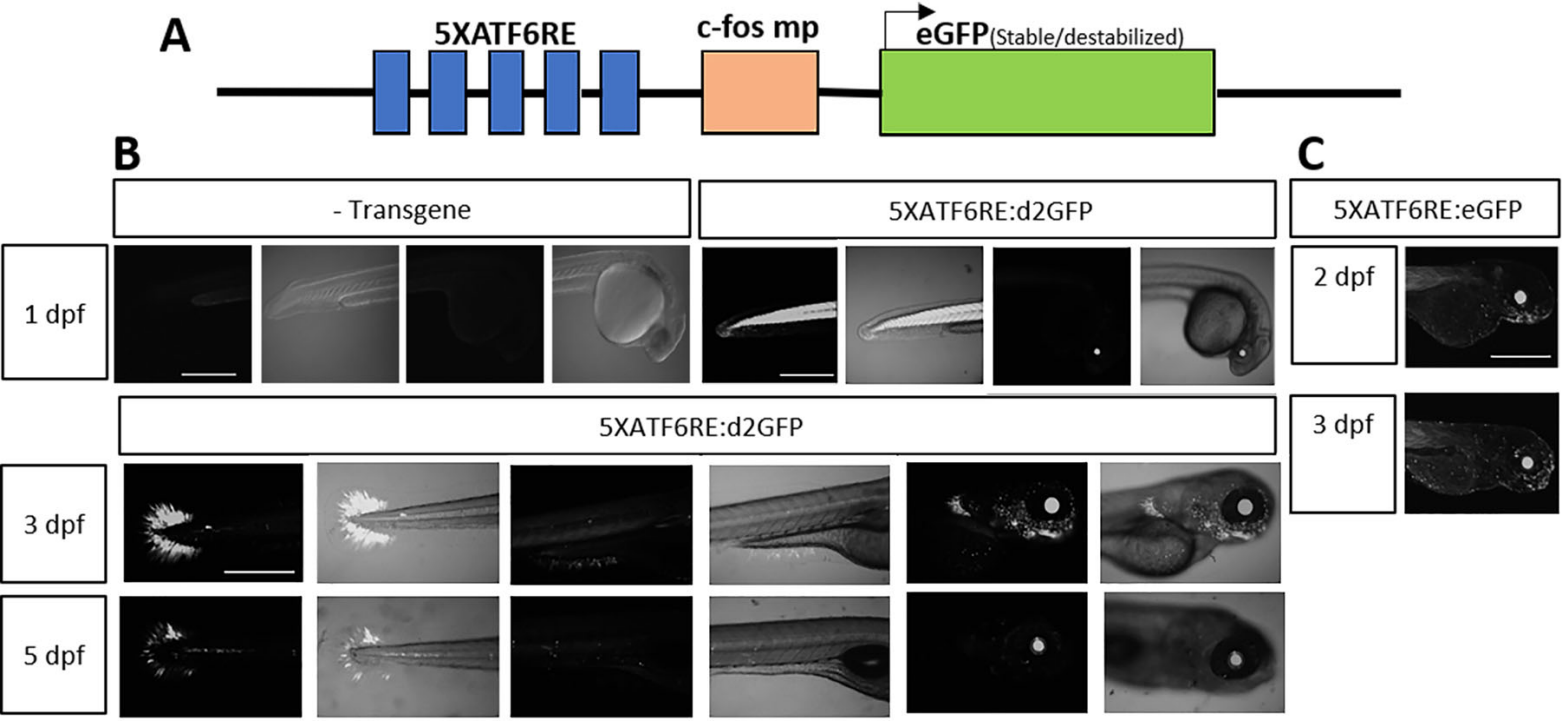
**Reporter expression in developing** **zebrafish.** (A) Schematic of the *ATF6* reporter plasmid showing five multimerized ATF6 binding sites (*5XATF6RE*) upstream of a *c-fos* minimal promoter (*c-fos mp*) driving expression of either destabilized GFP (*d2GFP*) or stable, enhanced GFP (*eGFP*). (B) Time-course analysis showing *5XATF6RE*:d2GFP expression at 1, 3 and 5 dpf. (C) Time-course analysis showing *5XATF6RE*:eGFP at 2 and 3 dpf. Scale bars: 1 mm.

**Fig. 2.**
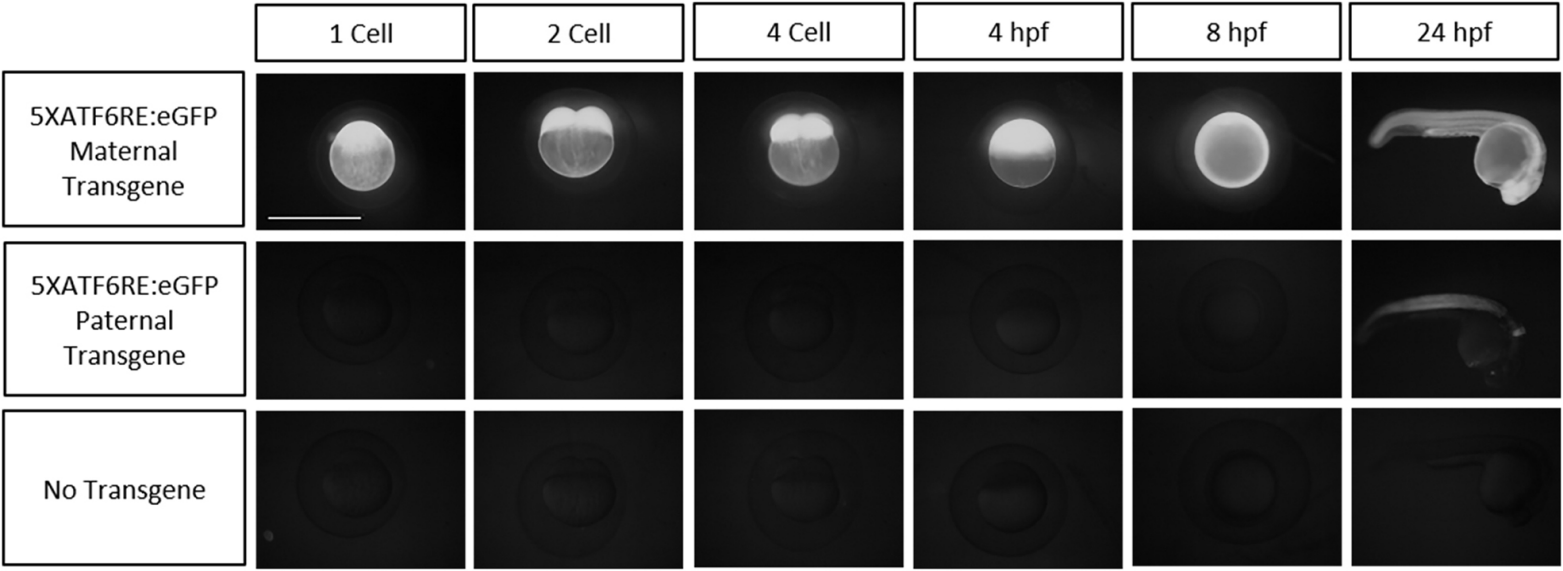
**Maternal/paternal inheritance of eGFP from the *5XATF6RE*:eGFP transgene.** Epifluorescent images showing embryos in which the *5XATF6RE:*eGFP was inherited maternally (top row) or paternally (middle row). Age-matched embryos not harboring a transgene are shown as an autoflourescence control (bottom row). Scale bar: 1 mm.

Studies characterizing Atf6 activity in animals have relied on nuclear immunolocalization and expression analysis of target genes (Samali et al., 2010). These strategies have been employed in zebrafish; for example, to study liver biology (Cinaroglu et al., 2011; Howarth et al., 2014). However, there are no current strategies to directly measure *ATF6* activity in intact animals. Therefore, we generated transgenic reporters to quantitate *ATF6* transcriptional activity in zebrafish. The transgenes use a previously identified *ATF6* consensus binding site (Wang et al., 2000) multimerized five times upstream of a minimal *c-fos* promoter driving either *eGFP* or *d2GFP* (*5XATF6RE:*GFP; Fig. 1A). By injecting the reporter plasmid into multiple zebrafish embryos, a consistent expression pattern was observed, demonstrating that, regardless of the transgene insertion site, the activity pattern was consistent (Fig. S1). *5XATF6RE:*d2GFP (Fig. 1B) and *5XATF6RE:*eGFP (Fig. 1C) transgene expression was observed highest in the lens and skeletal muscle. *5XATF6RE:*d2GFP expression was more dynamic (Fig. S2), consistent with a higher turnover rate through proteasomal targeting by a PEST domain. Skeletal muscle expression was highest at 1 day post-fertilization (dpf) and decreased over time, whereas lens expression stayed relatively consistent with age. Furthermore, dynamic expression was also observed in the caudal and pectoral fins, gills and brain through development (Fig. 1B). Interestingly, maternally inherited *5XATF6RE*-derived eGFP protein resulted in ubiquitous fluorescence throughout embryonic development (Fig. 2). The destabilized transgenic version, *5XATF6RE:*d2GFP, did not show this maternal effect, suggesting that the main source of fluorescence with the stable eGFP version is due to protein inheritance and perdurance, and not significant mRNA translation prior to zygotic expression. Because of the higher baseline fluorescence in embryos derived from maternal transgenes, paternally provided transgenic embryos were analyzed in subsequent experiments.

To quantitatively analyze *ATF6* reporter expression, the number of active transgene inserts was investigated. F1 zebrafish were outcrossed, and the number of F2 embryos with reporter expression were counted. With one copy of active transgene, ∼50% of F2 embryos are expected to have reporter expression, which was observed in *5XATF6RE:*d2GFP and *5XATF6RE:*eGFP F2 embryos (Fig. S3A). Additionally, the lens expression at 2 dpf was consistent between sibling embryos, demonstrating that endogenous expression of the transgene can be used for quantitative analysis (Fig. S3B,C).

### Characterization of transgene specificity

**Fig. 3.**
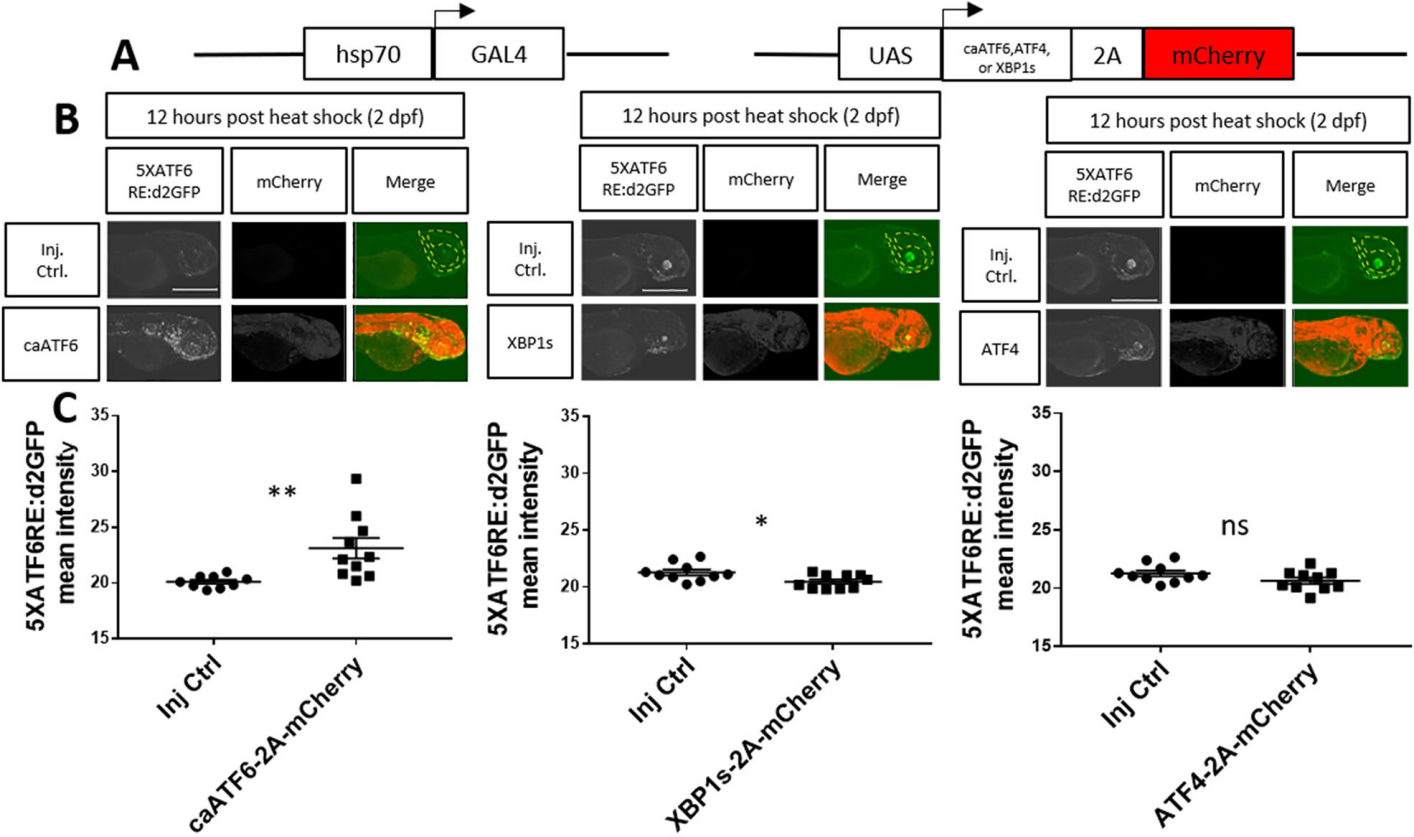
**Heat-shock-induced constitutive active ATF6 specifically induces reporter expression.** (A) Schematic of the *hsp70*:GAL4 construct used to induce ubiquitous expression of *UAS* constructs mosaically expressing either *caATF6*, *XBP1s* or *ATF4* with a *T2A* self-cleaving peptide and *mCherry*. (B,C) Representative images (B) and quantification of reporter expression (C) from embryos expressing *hsp70*:GAL4 and *5XATF6RE*:d2GFP transgenes that were injected with *mCherry*-tagged *UAS* plasmids to overexpress *caATF6* (left), *XBP1s* (middle) or *ATF4* (right) compared to injection control (Inj Ctrl) embryos injected with all components except overexpression plasmids. 2-dpf embryos were heat shocked and confocal images were captured 12 h later. Quantification of the head region (dashed line) revealed a significant increase in *5XATF6RE*:d2GFP intensity with *caATF6* overexpression (*P*=0.0067), a significant decrease with *XBP1s* overexpression (*P*=0.0182) and no significant change with *ATF4* overexpression (*P*=0.1055) compared to the injection control. *P≤0.05; **P≤0.01; ns, *P*>0.05; unpaired Student's *t*-test. All error bars are s.e.m. Scale bars: 1 mm.

**Fig. 4.**
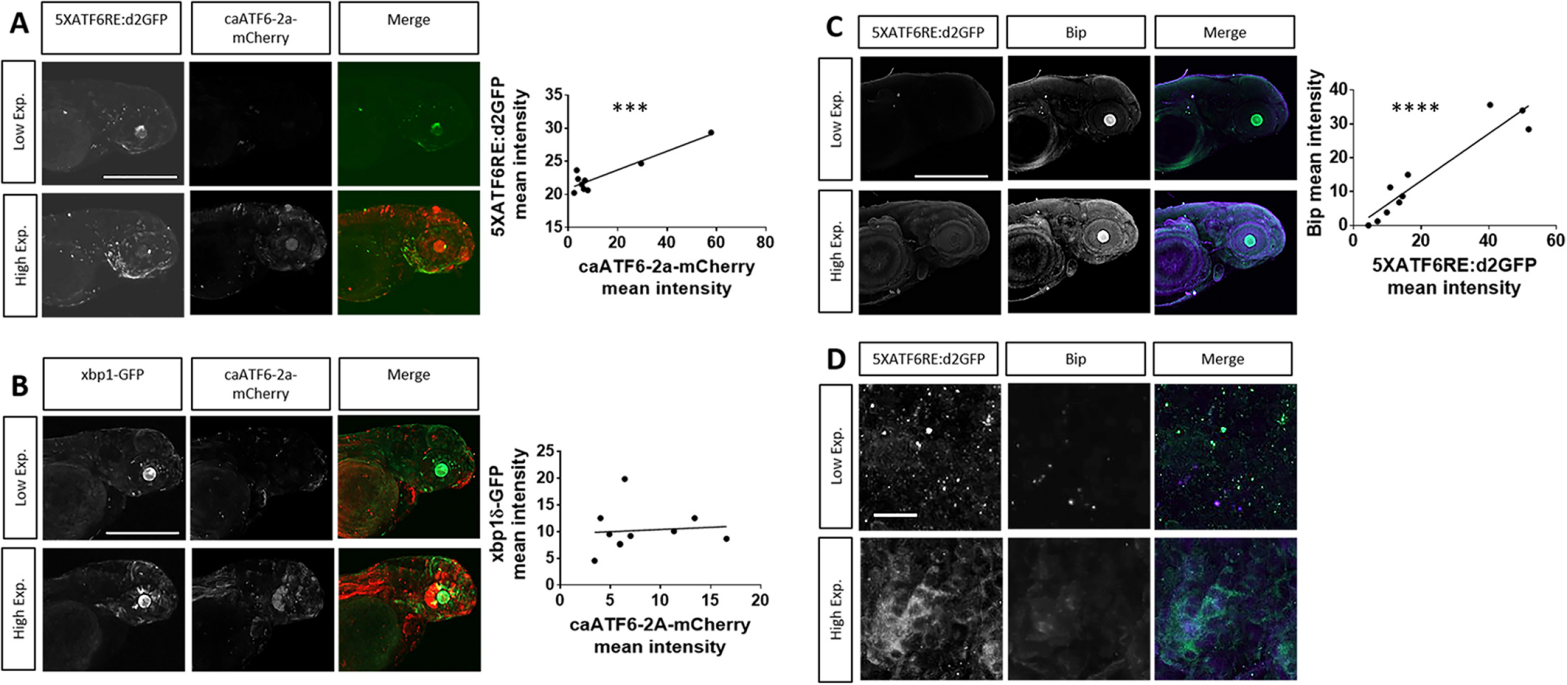
**The ATF6 target gene, *Bip*, is highly expressed with high *ATF6* reporter expression, while XBP1 expression does not correlate with caATF6.** (A-C) Embryos expressing *hsp70*:GAL4 and *5XATF6RE*:eGFP transgenes that were injected with an *mCherry*-tagged *UAS* construct to mosaically overexpress *caATF6.* 2-dpf embryos were heat shocked and confocal images were captured 12 h later live (A,B) or after fixation and staining (C,D), and mean intensity was quantified. (A) *5XATF6RE*:d2GFP expression positively correlates with *caATF6-2a-mCherry* expression (R^2^=0.8439, *P*=0.0005). (B) *xbp1δ-gfp* expression does not significantly correlate with *caATF6-2a-mCherry* expression (R^2^=0.0077, *P*=0.8099). (C) 12 h post-heat shock, embryos were fixed and stained for Bip (blue) or GFP (green). Bip expression positively correlates with *5XATF6RE*:d2GFP expression (R^2^=0.8899, *P*<0.0001). (D) 60× representative images showing low versus high cellular expression patterns of Bip and *5XATF6RE*:d2GFP. ***P<0.001; ****P<0.0001; Pearson's correlation coefficient. Scale bars: 1 mm (A-C); 0.02 mm (D).

Previously, a consensus binding site was defined for ATF6 in HeLa cells (Wang et al., 2000). Here, we sought to confirm and further investigate binding site specificity in zebrafish. Towards this goal zebrafish embryos expressing *5XATF6RE:*d2GFP and *hsp70:*GAL4 were injected with plasmids encoding active versions of transcription factors for each of the ER stress pathways (Fig. 3A). Zebrafish embryos were heat shocked at 2 dpf to induce robust, but mosaic, levels of each transcription factor. After imaging 12 h post-heat shock, constitutive active ATF6 (*caATF6*) caused significantly higher *5XATF6RE:*d2GFP expression compared to the injection control (*P*=0.0067; Fig. 3B,C). Conversely, heat-shock-induced expression of spliced XBP1 (*XBP1s*) slightly, but significantly, inhibited *5XATF6RE:*d2GFP expression (*P*=0.0182; Fig. 3B,C), and *ATF4* had no significant effect (*P*=0.1055; Fig. 3B,C). Additionally, induced *caATF6* expression also activated the *5XATF6RE:*eGFP transgene (*P*=0.0071; Fig. S4). *caATF6* also correlated with *5XATF6RE:*d2GFP expression (R^2^=0.8439, *P*=0.0005; Fig. 4A), but did not correlate with *xbp1δ-gfp* (Li et al., 2015) expression (R^2^=0.0077, *P*=0.8099; Fig. 4B). Furthermore, Bip expression significantly correlated with *5XATF6RE:*d2GFP expression (R^2^=0.8899, *P*<0.0001; Fig. 4C), suggesting that endogenous ATF6 target genes are upregulated with increased *ATF6* reporter expression. The *caATF6* expression resulted in autonomous transgene activation, as cells expressing *caATF6-2A-mCherry* colocalized with *5xATF6RE:*d2GFP significantly more than mCherry-only-expressing control cells (*P*=0.0005; Fig. S5). As 40% of d2GFP (ATF6-responsive cells) did not colocalize with mCherry (cells expressing caATF6), and 60% of mCherry-positive cells did not express detectible d2GFP, there may be evidence for non-autonomous expression and non-responsive cells, respectively (Fig. S5). Alternatively, discordant expression of fluorescent protein could reflect the possibility that transgenes preceding and following the 2A self-cleaving peptide were not 1:1 stoichiometrically expressed (Liu et al., 2017). Although ATF6 non-autonomy has not been previously described, Xbp1s has been shown to activate UPR in adjacent cells (Taylor and Dillin, 2013; Williams et al., 2014).

**Fig. 5.**
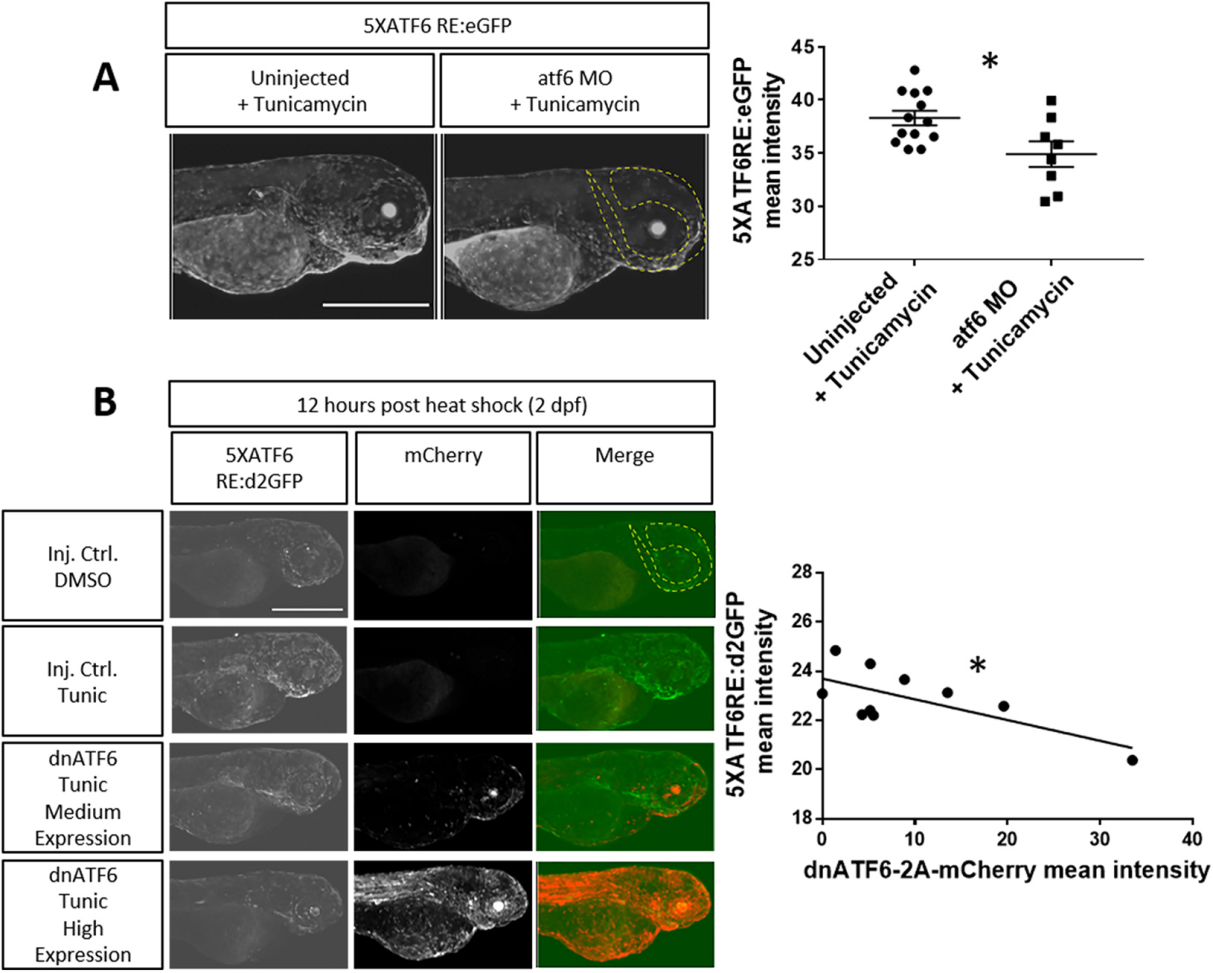
**Tunicamycin-induced reporter expression is blocked by inhibiting ATF6 translation and binding.** (A) After injection of an *atf6* morpholino (*atf6* MO) at the one- to four-cell stage to inhibit ATF6 translation, 1-dpf embryos were treated with Tunicamycin (Tunic) and expression at 3 dpf in the head region (dashed line) was quantified. *atf6*-MO-injected embryos had significantly less *5XATF6RE*:eGFP expression compared to tunicamycin-only-treated embryos (*P*=0.0150). Only embryos with detectable *5XATF6RE*:eGFP expression were analyzed. *P<0.05; unpaired Student's *t*-test. (B) Embryos expressing *hsp70*:Gal4 and *5XATF6RE*:d2GFP transgenes were injected with an *mCherry*-tagged *UAS* plasmid to mosaically overexpress dominant-negative ATF6 (*dnATF6*) that inhibits endogenous ATF6 binding. 2-dpf embryos were heat shocked, treated with tunicamycin, and expression of GFP and mCherry was quantified in the head region (dashed line) 12 h later. Representative images show that, compared to a DMSO-treated injection control, embryos with tunicamycin treatment show increased *5XATF6RE*:d2GFP expression, which decreases with increasing dnATF6-2A-mCherry expression, resulting in a significant negative correlation (R^2^=0.4712, *P*=0.0284). *P≤0.05; Pearson's correlation coefficient. All error bars are s.e.m. Scale bars: 1 mm.

To further explore reporter specificity, we conducted Atf6 loss-of-function experiments. Two approaches were employed: Atf6 protein levels were diminished using an *atf6*-translation-blocking morpholino (Cinaroglu et al., 2011), while Atf6 activity was inhibited by expression of a dominant-negative ATF6 (*dnATF6*). Using these reagents, we tested whether *5XATF6RE*:d2GFP expression could be blocked following induction by tunicamycin. Tunicamycin inhibits N-linked glycosylation at the ER and is commonly used to induce the UPR (Wang et al., 2000). We found that injection of *atf6* morpholino significantly blocked tunicamycin-induced *5XATF6RE*:eGFP expression (*P*=0.0150, Fig. 5A). Heat-shock-induced expression of *dnATF6* was variable, but the highest levels of dnATF6, as measured by mCherry intensity, completely negated *5XATF6RE*:d2GFP expression caused by tunicamycin treatment, resulting in a significant inverse correlation between dose of the dnATF6 and activation of the reporter (R^2^=0.4712, *P*=0.0284; Fig. 5B). Cumulatively, these gain- and loss-of-function experiments indicate that *ATF6* reporter activity is specific for ATF6, and not responsive to other UPR pathways.

### Monitoring *ATF6* reporter expression after induction of ER stress

**Fig. 6.**
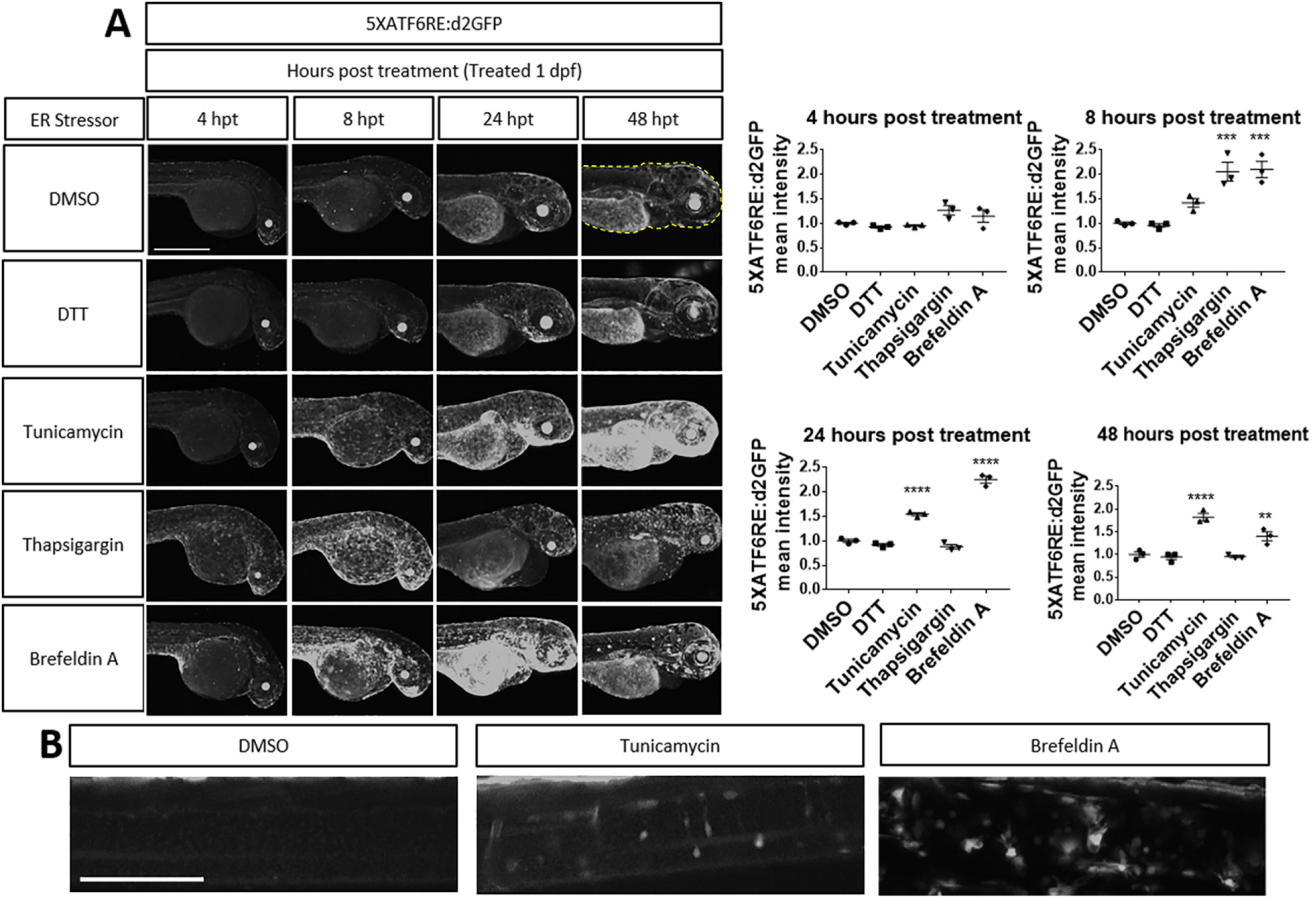
**Chemical ER stressors activate reporter expression.** (A) Zebrafish embryos were treated with ER stressors at 1 dpf. Quantification (dashed line) revealed that *5XATF6RE*:d2GFP expression was significantly higher at 8 hpt with thapsigargin (*P*=0.0004) and brefeldin A (*P*=0.0003) treatment, at 24 hpt with tunicamycin (*P*=0.0001) and brefeldin A (*P*=0.0001) treatment, and at 48 hpt with tunicamycin (*P*=0.0001) and brefeldin A (*P*=0.0065) treatment. **P≤0.01; ***P≤0.001; ****P≤0.0001; unpaired one-way ANOVA with Dunnett's post-hoc test with respect to DMSO control group. All error bars are s.e.m. (B) Representative images showing increased *5XATF6RE*:d2GFP expression in the spinal cord of embryos (at 48 hpt) treated with tunicamycin or brefeldin A compared to the DMSO-treated control. Scale bars: 1 mm (A); 0.1 mm (B).

The specific mechanism for how UPR is activated often results in differential pathway utilization. To probe how different chemical inducers of the UPR affect *ATF6* activation, we bath applied compounds to transgenic zebrafish embryos. Zebrafish embryos respire through their skin until 7 dpf (Rombough, 2002), and chemicals applied to the water are readily absorbed. After treatment of zebrafish embryos with a variety of established inducers of UPR, *5XATF6RE:*d2GFP expression was increased for most, but not all, compounds compared to dimethyl sulfoxide (DMSO)-treated control embryos (Fig. 6). Interestingly, for those activating the *ATF6* reporters, the dynamics and tissue responses were distinct for each chemical inducer of ER stress. Unexpectedly, dithiothreitol (DTT) did not activate the *ATF6* reporter transgenes. DTT is a strong reducing agent that can denature proteins by preventing intra- and intermolecular disulfide bonds. In contrast to DTT-mediated activation of *xbp1* (Li et al., 2015), DTT did not alter *5XATF6RE:*d2GFP expression compared to DMSO-treated control embryos (Fig. 6A). Tunicamycin, as noted previously, did induce reporter expression in a widespread manner, with peak levels at 48 h post-treatment (hpt). Thapsigargin is an inhibitor of the ER Ca^2+^ ATPase, resulting in calcium efflux and dysfunction of the organelle. Thapsigargin-induced expression of *5XATF6RE*:d2GFP peaked at 8 hpt and subsequently decreased by 24 hpt (Fig. 6A). Brefeldin A inhibits vesicle formation at the Golgi and ultimately results in fusion of Golgi and ER membranes, leading to ER stress. Brefeldin A induced expression of *5XATF6RE*:d2GFP primarily in the yolk and gills, with a peak at 24 hpt (Fig. 6A). To further analyze differential *ATF6* activation in response to ER stressors, we more closely inspected spinal cord neurons (Fig. 6B). Treatment with tunicamycin or brefeldin A, but neither DMSO nor DTT, activated the *ATF*6 reporter in a distinct subset of cells. Together, this analysis shows that the *ATF6* activity reporters respond to a variety of ER stressors, but suggests that distinct mechanisms of ER insult result in differential response kinetics and tissue sensitivities. Like *5XATF6RE*:d2GFP, *5XATF6RE*:eGFP expression levels were also elevated after treatment with tunicamycin, thapsigargin and brefeldin A, but not DTT or DMSO (Fig. S6).

**Fig. 7.**
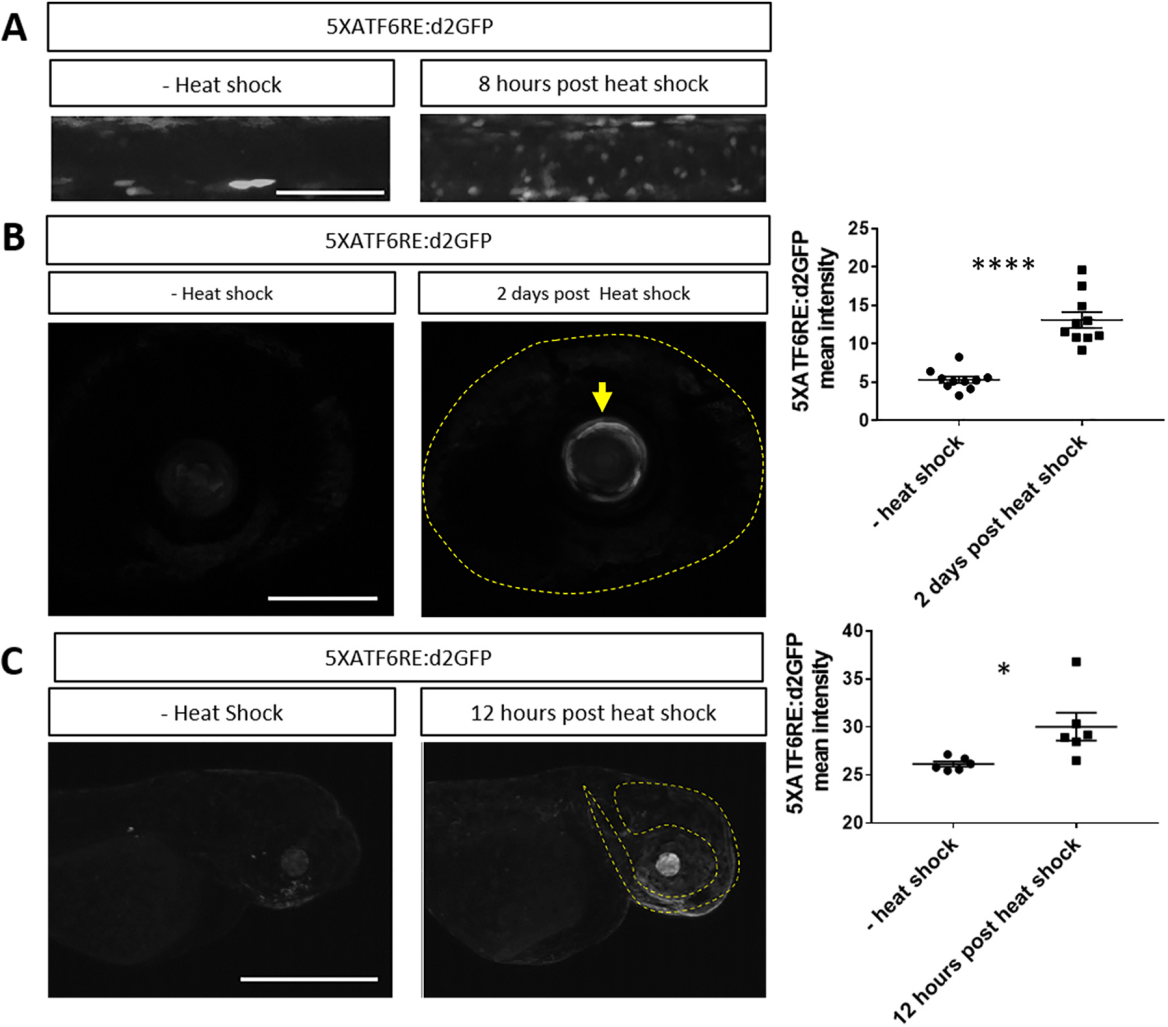
**Heat shock activates reporter expression.** (A) Representative images showing increased *5XATF6RE*:d2GFP expression in the spinal cord 8 h after heat shock compared to a control without heat shock. (B) Representative images and quantification of the lens (indicated by an arrow) revealed significantly higher levels of *5XATF6RE*:d2GFP expression 2 days post-heat shock compared to a control without heat shock (*P*<0.0001). The dashed line outlines the eye. (C) Representative images and quantification of the head region (dashed line) revealed significantly higher levels of *5XATF6RE*:d2GFP expression 12 h post-heat shock compared to a control without heat shock (*P*=0.0248). *P≤0.05; ****P≤0.0001; unpaired Student's *t*-test. All error bars are s.e.m. Scale bars: 0.1 mm (A,B); 1 mm (C).

In addition to chemical stress, heat stress can be used to alter proteostasis (Long et al., 2012) and can induce apoptosis at 8 h post-heat shock in the spinal cord (Yabu et al., 2001). We hypothesized that *ATF6* expression might also be induced by heat stress. Indeed, 8 h after heat shock, *5XATF6RE:*d2GFP reporter expression was elevated in the spinal cord (Fig. 7A). Similarly, reporter expression was significantly higher in the lens (*P*<0.0001; Fig. 7B) and head region (*P*=0.0248; Fig. 7C) at various times post-heat shock. Therefore, *ATF6* transcriptional activity is elevated in multiple tissues in response to altered proteostasis.

### Investigation of ATF6 in neurodegenerative disease

**Fig. 8.**
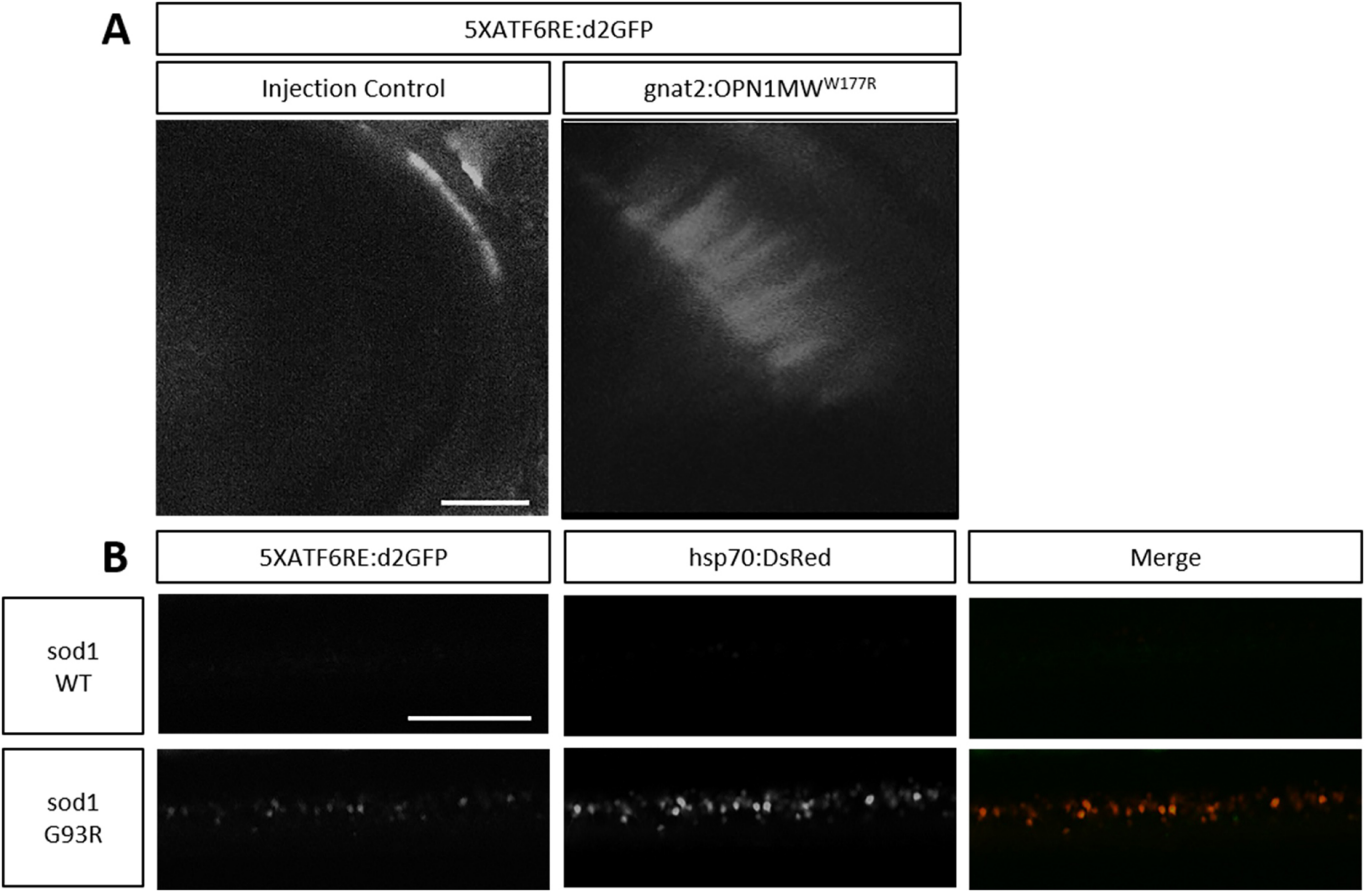
**Reporter expression is activated in ALS and cone dystrophy zebrafish disease models.** (A) Representative images showing that embryos injected with *gnat2:*OPN1MW^W177R^ mutant opsin have elevated *5XATF6RE*:d2GFP expression in photoreceptors, which is absent in injection control embryos injected with all components except the overexpression construct. (B) Representative images showing overlap of *hsp70*:DsRed and *5XATF6RE*:d2GFP expression in *sod1 G93R* mutant spinal cord, which is absent in WT *sod1* control spinal cord. Scale bars: 0.02 mm (A); 0.1 mm (B).

The results above suggest that eye and spinal cord tissue may be susceptible to altered proteostasis and ER stress. Many neurodegenerative diseases are characterized by misfolded proteins and UPR activation. Neurons, including photoreceptors, are highly metabolically active with a significant protein turnover demand and therefore could be more susceptible to ER stress. In fact, photoreceptors are the most metabolically active cell type in the human body (Sung and Chuang, 2010; Wong-Riley, 2010). Interestingly, in mice and humans, mutation to *ATF6* results in age-dependent photoreceptor degeneration (Ansar et al., 2015; Kohl et al., 2015). This result suggests an important protective effect of ATF6 in these photoreceptor cells. Therefore, we hypothesized that ATF6 signaling is responsive to misfolded proteins that accumulate at the ER in photoreceptors. When a misfolding-prone mutant cone opsin (OPN1MW^W177R^), which is retained in the ER (Gardner et al., 2010), was expressed in cone photoreceptors, there was a dramatic increase in *ATF6* reporter expression (Fig. 8A). These findings support previous work that indicated that ATF6 signaling is important for photoreceptor homeostasis and demonstrate the utility of the *ATF6* activity reporters in studying neurodegenerative disease.

ALS is another neurodegenerative disease characterized by misfolded proteins. Although a diverse group of genes result in ALS when mutated, a common pathology includes protein aggregation, activation of UPR and death of motor neurons (Matus et al., 2013). One of the most common genes mutated is superoxide dismutase (*SOD1*). Defects in SOD1 that result in ALS are not due to loss of redox control, but instead are due to toxic gain of function that increases the rate of protein misfolding (Bruijn et al., 1998; Wiedau-Pazos et al., 1996; Yim et al., 1996). Motor neurons with mutations to amino acid G93 of *SOD1* are particularly susceptible to misfolding (Stathopulos et al., 2003). Based on this observation, a zebrafish model of ALS was established by transgenic overexpression of *sod1 G93R* (McGown et al., 2013; Ramesh et al., 2010). Using a general cell stress reporter gene (*hsp70*:DsRed), McGown et al. (2013) demonstrated that mutant *sod1 G93R* zebrafish embryos had elevated stress in the spinal cord, which did not occur in transgenic zebrafish expressing wild-type (WT) *sod1*. Interestingly, these cells were identified as interneurons, mostly glycinergic interneurons, suggesting that this cell type might be particularly susceptible in disease progression at pre-symptomatic stages of ALS. ATF6 is elevated in *sod1 G93A* mice and in human ALS patients (Atkin et al., 2006, 2008; Prell et al., 2019). Therefore, we tested the role of ATF6 in an ALS zebrafish model expressing the *hsp70*:DsRed reporter. *5XATF6RE*:d2GFP colocalized with *hsp70*:DsRed expression in *sod1 G93R* zebrafish spinal cords, but both stress reporters were absent in WT *sod1* zebrafish (Fig. 8B). These results indicate that multiple protective mechanisms including chaperone expression and UPR are increased in stressed interneurons early in ALS disease progression. Ultimately, these observations suggest that neurons are susceptible to unfolded protein stress at the ER, and that ATF6 could be an important target in modifying neurological diseases.

## DISCUSSION

Investigation of Atf6 dynamics in whole animals has been limited to monitoring Atf6 immunoreactivity or target gene expression. Furthermore, in zebrafish, detailed studies of Atf6 have been conducted on the liver, but its role in other tissues has not been well characterized (Cinaroglu et al., 2011; Howarth et al., 2014). The development of transgenic zebrafish that report *ATF6* activity will facilitate studies across tissues and over time. Here we describe the establishment and validation of *ATF6*-responsive transgenic zebrafish. The transgene activation was specific for ca*ATF6* but not *ATF4*. Expression of XBP1s conferred a slight, but significant, decrease in *ATF6* reporter activity, suggesting that activation of XBP1-based UPR may confer a negative feedback on ATF6 signaling. Expression of caATF6, however, did not correlate with *xbp1δ-gfp* activity. ATF6 and XBP1 pathways have been shown to have both overlapping or distinct functions depending on the cellular context (Yoshida et al., 2001; Lee et al., 2002, 2003; Yamamoto et al., 2004; Shoulders et al., 2013). Therefore, additional experiments are warranted to investigate crosstalk of UPR pathways in various cell types using the *ATF6* reporters. The transgenes demonstrate dynamic activation during development, and the transgenic fish can be used to monitor *ATF6* activity in diseases characterized by ER stress and unfolded proteins. In development, *ATF6* reporters were expressed highest in the lens and skeletal muscle, but were also present in fins, the central nervous system and the branchial arch region. Elevated *ATF6* activity in the lens and muscle is consistent with those tissues expressing high levels of Atf6 during development (Firtina and Duncan, 2011; Nakanishi et al., 2005; Wang et al., 2015). Similar to maternal *xbp1δ-gfp* expression (Li et al., 2015), maternal *5XATF6RE:*eGFP expression was pronounced, suggesting increased demand on ER function during oogenesis and/or embryogenesis immediately following fertilization and thus a requirement for stress protection.

With regard to disease relevance, we showed that *ATF6* transgenes are responsive to cone photoreceptor expression of a variant opsin protein (OPN1MW^W177R^) that is known to misfold and cause photoreceptor degeneration in humans (Gardner et al., 2010). Although ER retention has been characterized with the mutant cone opsin, the cell stress response had not been previously characterized. Photoreceptors may be particularly sensitive to ER stress, as they synthesize and traffic tremendous amounts of phototransductive machinery as part of the daily shedding of their outer segments (Pearring et al., 2013). Supporting this idea are several observations. First, intravitreal injection of tunicamycin, a potent ER stress inducer and activator of *ATF6* as defined in our initial studies, results in severe photoreceptor degeneration (Alavi et al., 2015; Shimazawa et al., 2007). Furthermore, light-induced retina degeneration also shows signs of ER stress, suggesting that the UPR may be a common pathway activated in photoreceptor degeneration (Kroeger et al., 2012). Perhaps most significantly, mutations to *ATF6* cause achromatopsia and result in loss of cone photoreceptor cells (Kohl et al., 2015). Characterization of a wide array of *ATF6* mutant alleles suggests that patients have elevated risk to ER stress that is compounded by both ER retention of the mutant protein and absence of ATF6 function (Chiang et al., 2017). ATF6 is also known to be activated in ALS and other neurodegenerative diseases. For example, *ATF6* is activated in the spinal cord of ALS patients (Atkin et al., 2008), and *ATF6* expression is decreased following disruption of the ALS-causing *VAPB* gene (Chen et al., 2010; Nardo et al., 2011). Using a zebrafish model of ALS in which a disease allele of *sod1* (G93R) is expressed from the *sod1* promoter (Ramesh et al., 2010), we found elevated *ATF6* activity in spinal cord interneurons. These observations in both photoreceptors and spinal neurons suggest that modifying *ATF6* activity could be a therapeutic approach to mitigate neurodegenerative diseases. Indeed, de-repression of Atf6 in a mouse model of Huntington's disease provides neuroprotection to both striatal and hippocampal neurons (López-Hurtado et al., 2018; Naranjo et al., 2016). Furthermore, a small-molecule activator of ATF6 reduces amyloidogenic protein secretion and aggregation, providing additional justification for targeting the ATF6 pathway in neurodegenerative disease (Plate et al., 2016).

One unexpected observation from our experiments was that, in addition to autonomous activation of the ATF6 activity reporter (*5XATF6RE:*d2GFP) by expressing ATF6 protein (*caATF6-2A-mCherry*), there was also non-autonomous reporter response. Cells proximal to those expressing constitutively activated ATF6 were often marked by high d2GFP expression without detectible *caATF6-2A-mCherry* expression. We performed a time-course analysis of d2GFP expression at 3, 6, 9 and 12 h following induction of *caATF6-2A-mCherry* but never measured complete overlap in expression of mCherry and d2GFP, suggesting that the non-autonomy is not due to differential fluorescence turnover of either marker gene (data not shown). These findings are interesting in the context of work in *Caenorhabditis* *elegans* demonstrating that ER stress within neurons triggers autonomous *xbp1* activation that results in release of small clear vesicles. Signals packaged within the vesicles appear to trigger Xbp1-mediated UPR non-autonomously within peripheral tissues, which ultimately promotes stress-resistant longevity for the entire worm (Taylor and Dillin, 2013). Similarly, expression of *Xbp1s* in mouse hippocampal neurons leads to non-autonomous activation of ER stress in the liver (Williams et al., 2014). More detailed investigation into the mechanisms underlying ATF6 non-autonomy in zebrafish could be accomplished with the tools described here.

As an experimental resource to study homeostasis and disease, there are multiple potential uses for the *ATF6* reporter lines. Although small molecule screens have recently identified activators (Plate et al., 2016) and inhibitors (Gallagher and Walter, 2016) of the ATF6 pathway, additional targets of the pathway may prove beneficial. Zebrafish are particularly amenable for chemical-genetic screens, and having whole-animal readouts for *ATF6* activity provides a sophisticated platform for such assays. In addition to screens, the reporter lines will facilitate the analysis of cells actively responding to ER stress. For example, by sorting cells based on their fluorescence, transcriptomics, metabolomics or proteomics could reveal susceptible cell types and cell states. In addition, such analysis might also reveal other pathways co-activated/inhibited with *ATF6* induction. These and certainly other studies will shed more light on the mechanism and potential modulation of ATF6 signaling.

## MATERIALS AND METHODS

### Fish maintenance

Zebrafish (*Danio rerio*) were maintained at 28.5°C on a recirculating filtered water system (Aquatic Habitats, Apopka, FL, USA) in reverse-osmosis-purified water supplemented with Instant Ocean salts (60 mg/l) on a 14-h light: 10-h dark lighting cycle and fed a standard diet (Westerfield, 1995). All animal husbandry and experiments were approved and conducted in accordance with the guidelines set forth by the Institutional Animal Care and Use Committee of the Medical College of Wisconsin.

### Generation of plasmids

*ATF6RE:*eGFP and *ATF6RE:*d2GFP reporter plasmids with a *c-fos* minimal promoter were amplified by Genescript from Addgene plasmid 11976 (Wang et al., 2000), which contains the *ATF6* response element TGACGTGGCGATTCC interposed by linker sequences and multimerized five times (*5XATF6RE*). The purified *5XATF6RE* was placed into a 5′ entry clone and the three-part Gateway system (Thermo Fisher Scientific, Waltham, MA, USA) was used to assemble the 5′ *5xATF6* response element in front of a *c-fos* minimal promoter followed by either *eGFP* or *d2GFP* and a 3′ *polyA* tail. The backbone vector containing Tol2-inverted repeats flanking the transgene constructs was used to facilitate plasmid insertion into the zebrafish genome (Fig. 1A; Kawakami et al., 2000; Kwan et al., 2007). Similarly, middle-entry plasmids used for overexpression experiments were made by PCR amplification from parent plasmids, followed by Gateway recombineering using an upstream-activator sequence (*UAS*) promoter and a downstream *T2A* self-cleaving peptide fused to *mCherry*, to mark expressing cells. Parent and final plasmids are listed in Table S1. Overexpression experiments using each plasmid were accomplished via the *GAL4/UAS* system (Scheer and Campos-Ortega, 1999) using *hsp70:*GAL4-expressing zebrafish for inducible, ubiquitous expression after heat shock at 39°C for 1 h. For experiments, embryos were injected at the one- to four-cell stage with 9.2 nl of working solution containing 10 ng/µl construct and 5 ng/µl transposase. To construct the OPN1MW^W177R^ plasmid, the OPN1MW^W177R^ sequence was PCR amplified, inserted into a middle-entry vector, and then added to a Tol2 destination vector downstream of a cone-photoreceptor-cell-specific promoter (*gnat2*) using the Gateway system.

### Microinjection and generation of transgenic ATF6 reporter zebrafish

Transposase mRNA was injected with *ATF6RE:*eGFP or *ATF6RE:*d2GFP plasmid DNA to generate F0 transgenic lines. F0 fish were analyzed to ensure consistent transgene expression regardless of the integration site. F0 injected fish were raised to adulthood and outcrossed to wild-type zebrafish to establish four separate F1 ATF6 reporter zebrafish with germline integration of the transgene. F1 transgenic larvae were raised to adulthood and 16 total fish from four F1 lines were outcrossed to wild-type zebrafish for expression analysis (Fig. S1). F2 zebrafish transgenic lines containing one active copy of each transgene were used for all subsequent analysis. These lines are designated Tg(Hsa.*ATF6RE:*eGFP)mw84 or Tg(Hsa.*ATF6RE:*d2GFP)mw85.

### Chemical and heat shock stress in zebrafish embryos

Chemical induction of ER stress in zebrafish embryos was accomplished by using a final concentration of 0.5 mM DTT (NEB #B1034S; Invitrogen Y00147), 1 µM thapsigargin (Sigma-Aldrich #T9033), 1 µg/ml tunicamycin (Sigma-Aldrich #T7765), and 5 µM brefeldin A (Sigma-Aldrich #B7651) in 10 ml phenylthiourea (PTU) in a 16 mm×50 mm Petri dish. For heat stress, Petri dishes containing embryos in 5 ml PTU and sealed using parafilm were incubated in a water bath set to 39°C for 1 h.

### Morpholinos

*atf6* ATG morpholino, 5′-ACATTAAATTCGACGACATTGTGCC-3′ (Cinaroglu et al., 2011), was synthesized by GeneTools; 9.2 nl of a working solution containing 700 µM morpholino was injected into zebrafish embryos at the one- to four-cell stage.

### Antibodies

Anti-Bip was purchased from Sigma-Aldrich (rabbit polyclonal, #G9043) and used at 1:100 dilution for whole-mount staining. Validation in zebrafish has previously been demonstrated (Niu et al., 2012).

### Image acquisition and analysis

For maternal contribution experiments, a Leica MZ FLIII epifluorescent stereomicroscope was used for image acquisition. *ATF6* reporter activity was observed with a Nikon Eclipse E600FN confocal system (Nikon, Tokyo, Japan) using the Nikon EZ-C1 software and a 10× air, 0.3 NA or a 40× water, 0.8 NA objective (Nikon Instruments). Reporter expression was quantified using ImageJ (National Institutes of Health, Bethesda, MD, USA). For tissue-specific quantification, a region of interest (ROI) was drawn around the site of interest. A maximum-pixel-intensity projection image from a *z*-stack containing the total fluorescence in the ROI was analyzed for mean pixel intensity without background subtraction or normalization. For colocalization analysis, Imaris 3D Coloc software was used (BitPlane).

### Statistical analysis

Mean pixel intensity measurements of reporter expression were processed using Excel (Microsoft, Redmond, WA, USA) and graphed using Prism (GraphPad, La Jolla, CA, USA). An unpaired, two-tailed Student's *t*-test was used to analyze graphs with two groups. For three or more groups, a one-way ANOVA was conducted with Dunnett's post-hoc analysis for pairwise comparisons.

## Supplementary Material

Supplementary information
