# Supplementary material for: Establishment and validation of an endoplasmic reticulum stress reporter to monitor zebrafish ATF6 activity in development and disease
Source: Dis Model Mech. 2020 Jan 28;13(1):dmm041426. doi: 10.1242/dmm.041426 (PMC6994954; doi:10.1242/dmm.041426)
Supplement: Supplementary information [file dmm-13-041426-s1.pdf]

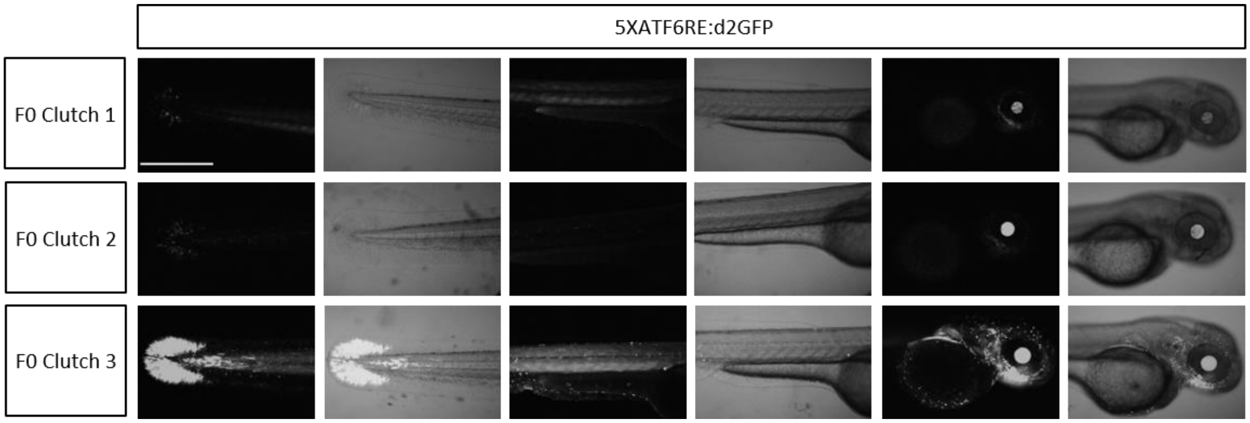

Figure S1. Transient reporter expression is consistent regardless of copy number and insertion site. Representative images showing mosaic expression patterns in three separate clutches injected with *5XATF6RE:d2GFP* plasmid DNA. Scale bar=1mm.

Disease Models & Mechanisms • Supplementary information

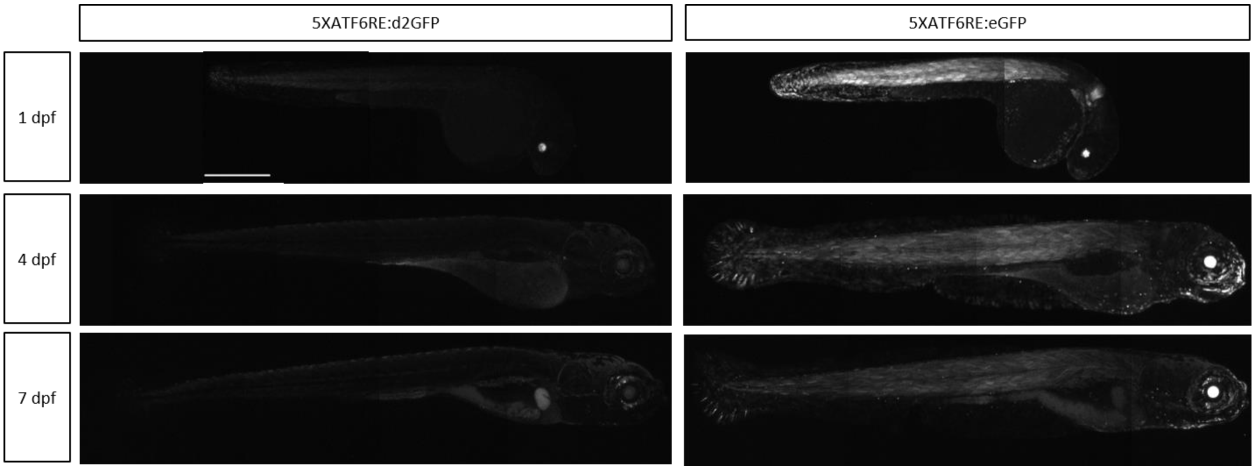

Figure S2. Reporter expression dynamics throughout development. Representative images showing expression of one active copy of *5XATF6RE:d2GFP* and *5XATF6RE:eGFP*. The microscope settings were kept consistent across groups and throughout the timecourse. Scale bar=1mm.

Disease Models & Mechanisms • Supplementary information

A

Name	+ Expression	- Expression	Total	% expressing
5XATF6RE:d2GFP	46	72	118	40
5XATF6RE:eGFP	34	31	65	52

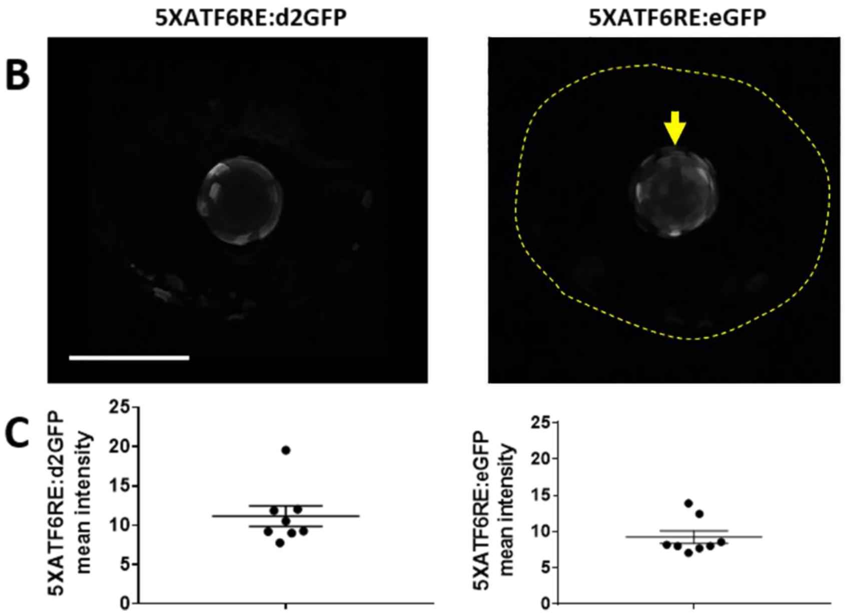

Figure S3. Single active transgene expression is consistent across embryos. (A) Number of F2 embryos expressing *5XATF6RE*:d2GFP or *5XATF6RE*:eGFP after an F1 outcross. (B,C) Representative images (B) and quantification (C) of reporter expression in the lens (arrow) from 2 dpf F2 generation embryos from an F1 outcross. The dotted line outlines the eye. Scale bar=0.1mm.

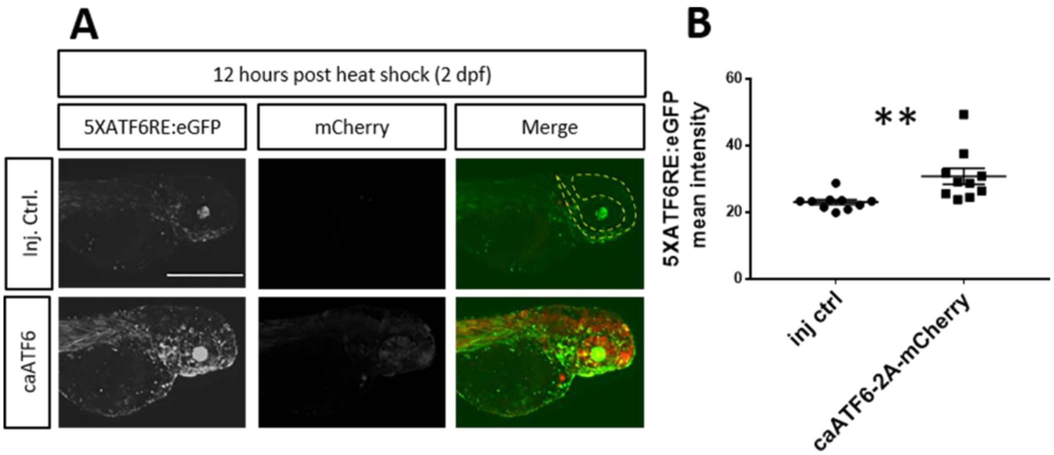

Figure S4. eGFP reporter expression is increased after heat shock induced constitutive active ATF6 expression. (AB) Representative images (A) and quantification of reporter expression (B) from embryos expressing *hsp70*:GAL4 and *5XATF6RE*:eGFP transgenes that were injected with an *mCherry* tagged *UAS* construct to mosaically overexpress *caATF6* compared to injection control (Inj. Ctrl.) embryos injected with all components except overexpression constructs. 2dpf embryos were heat shocked and confocal images were captured 12 hours later. Quantification (dotted line) revealed a significant increase in *5XATF6RE*:eGFP expression with overexpression of *caATF6* compared to the injection control (p=0.0071). ** p≤0.01; unpaired t-test. Scale bar=1mm.

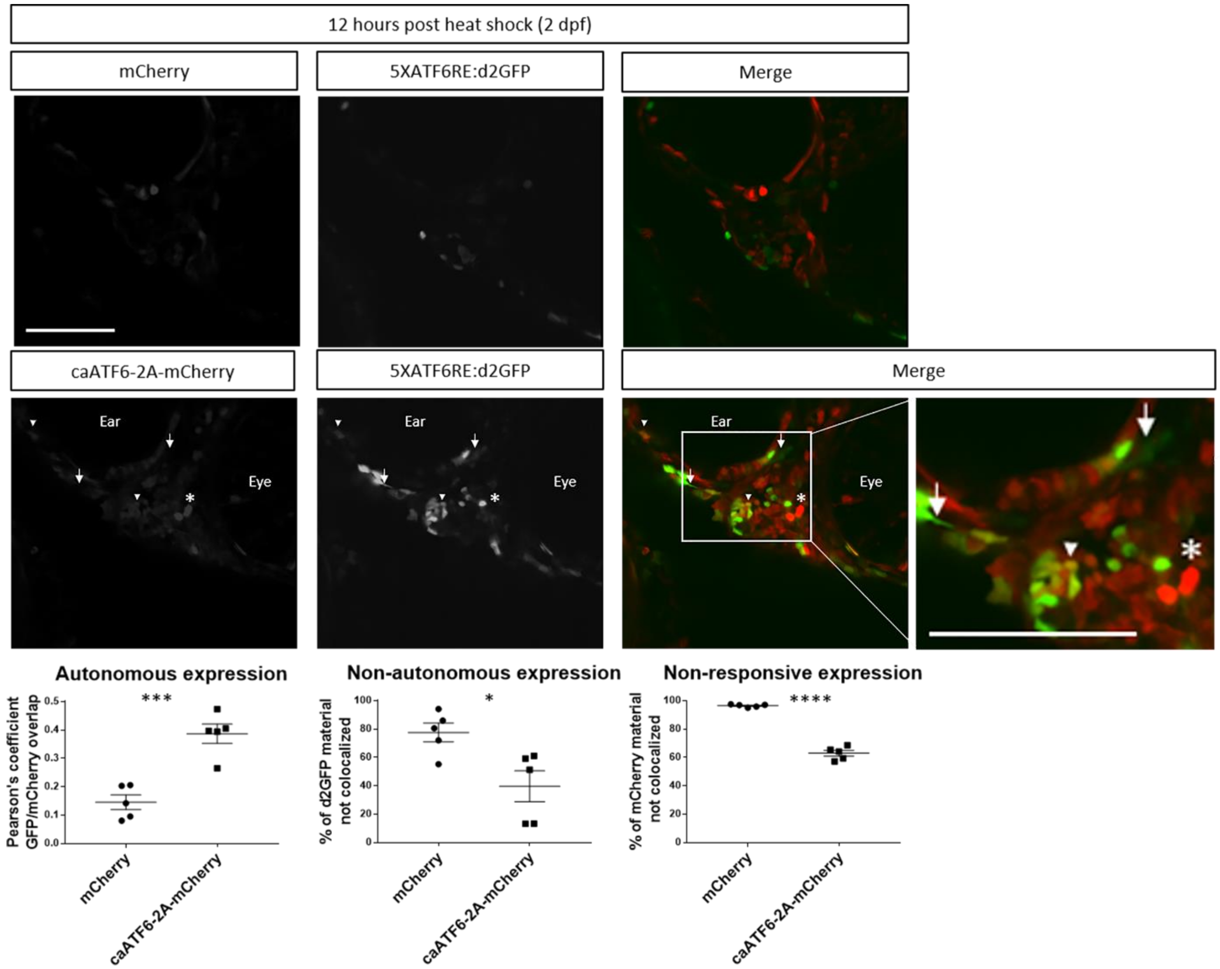

Figure S5. Autonomous reporter activation by constitutive active ATF6. Embryos expressing *hsp*70:GAL4 and *5XATF6RE*:d2GFP transgenes were injected with an *mCherry* tagged *UAS* construct to mosaically overexpress *caATF6* or a *UAS* construct with *mCherry* expression alone as a control. 2 dpf embryos were heat shocked and confocal images zoomed on the jaw region where reporter expression was highest were captured 12 hours later. Quantification revealed that compared to *mCherry* expression alone, caATF6-2A-mCherry has significantly higher autonomous expression ($p=.0005$; arrowheads) and significantly lower non-autonomous expression ($p=0.0177$; arrow) and non-responsive expression. ($p<0.0001$; asterisk). * $p\leq0.05$; ***$p\leq0.001$; ****$p\leq0.0001$; unpaired t-test. Scale bars=0.1mm.

Disease Models & Mechanisms • Supplementary information

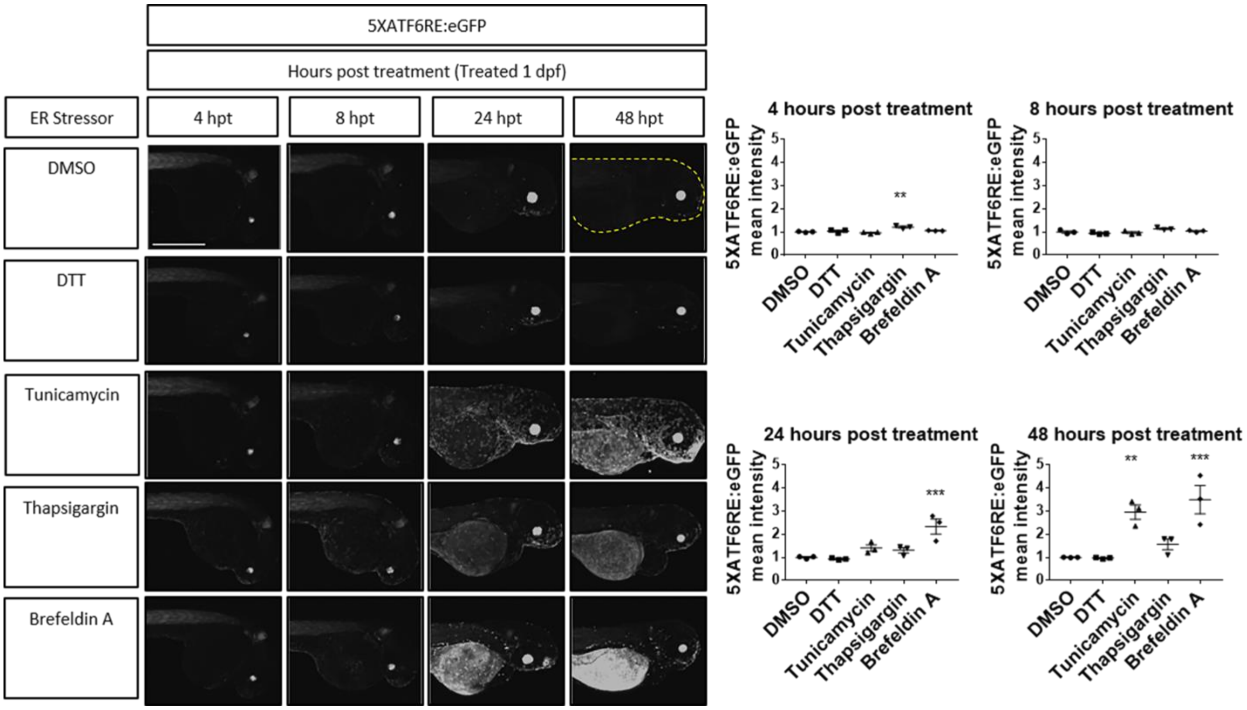

Figure S6. Chemical ER stressors activate eGFP reporter expression. (A) Zebrafish embryos were treated with ER stressors at 1 dpf. Quantification (dotted line) revealed that *5XATF6RE*:eGFP expression was significantly higher at 4 hpt with Thapsigargin (p=0.0012) treatment, 24 hpt with Brefeldin A (p=0.0008) treatment, and 48 hpt with Tunicamycin (p=0.0054) and Brefeldin A (p=0.0010) treatment. ** p≤0.01; *** p≤0.001; unpaired one-way ANOVA with Dunnett's post-hoc test with respect to DMSO control group. Scale bar=1mm

Disease Models & Mechanisms • Supplementary information

Table S1: Parent and final plasmids

Plasmid Name	Parent Plasmid	Final Plasmid
ATF6 constitutive active (caATF6) (1-373)	Adgene plasmid 27173, pCGN-ATF6 (1-373)	Tol2-UAS: caATF6 (1-373)-2a-mcherry
ATF6 dominant negative (dnATF6) (171-373)	Adgene plasmid 27173, pCGN-ATF6 (1-373)	Tol2-UAS: dnATF6 (171-373)-2a- mcherry
ATF4 full length	Adgene plasmid 82190, pDonr223_ATF4_WT	Tol2-UAS: ATF4-2a-mcherry
Spliced XBP1 (XBP1s)	Adgene plasmid 63680, pCMV5-Flag-XBP1s	Tol2-UAS: XBP1s-2a-mcherry
